# A Rare Case of Hunter Syndrome (Mucopolysaccharidosis II) With Bilateral Maculopathy Associated With Rod-Cone Dystrophy

**DOI:** 10.7759/cureus.93034

**Published:** 2025-09-23

**Authors:** Adjoa Safoa Panyin Quaicoe, Elisa E Cornish, Robert Chong

**Affiliations:** 1 Department of Ophthalmology, Emmanuel Eye Medical Centre, Accra, GHA; 2 Ophthalmology, Save Sight Institute, University of Sydney, Sydney, AUS; 3 Department of Ophthalmology, Sydney and Sydney Eye Hospital, Sydney, AUS; 4 Department of Ophthalmology, Westmead Hospital, Sydney, AUS

**Keywords:** case report, hunter syndrome, maculopathy, mucopolysaccharidosis, rod-cone dystrophy

## Abstract

Hunter syndrome is an X-linked recessive lysosomal storage disorder that is caused by a mutation in the iduronate sulfatase gene. Both anterior and posterior segment abnormalities are found as a result of the accumulation of glycosaminoglycans in ocular tissues. Retinal dystrophy, particularly rod-cone dystrophy, has a major effect on visual acuity, leading to significant visual impairment as the condition worsens. We report a case of a 53-year-old male patient of Asian descent previously diagnosed with Hunter syndrome, who presented with progressive difficulty in visual tracking and colour recognition. Fundus examination revealed bull's eye maculopathy in both eyes. Optical coherence tomography revealed severe attenuation of the outer retinal layers at the macula. Electrophysiological tests showed reduced photopic and scotopic responses, with P50 responses severely attenuated, and visual field testing showed a central scotoma in both eyes. Patients with Hunter syndrome can present with retinitis pigmentosa or rod-cone dystrophy. Accumulation of glycosaminoglycans in the retinal pigment epithelium results in photoreceptor loss, affecting both rods and cones. Maculopathy associated with rod-cone dystrophy may be associated with this condition.

## Introduction

Mucopolysaccharidoses are a group of inherited lysosomal storage disorders that are caused by intracellular and extracellular accumulation of glycosaminoglycans (GAGs). Hunter syndrome, which is inherited as an X-linked recessive syndrome, is caused by a mutation in the iduronate sulfatase (IDS) gene. It is also known as mucopolysaccharidosis (MPS) II and is characterized by a deficiency of the enzyme iduronate 2-sulfatase, which results in the progressive accumulation of dermatan sulfate and heparan sulfate [1].

The global incidence of MPS II is one in 162000 live male births [2]. The onset of symptoms usually occurs at the preschool age [2,3]. MPS II is a multi-organ syndrome that may affect the central nervous system, respiratory system, cardiovascular system, gastrointestinal system, and the musculoskeletal system. Thus, it may manifest as structural abnormalities such as short stature, hydrocephalus, upper airway obstruction, hepatosplenomegaly, and valvular heart disease [2]. Ocular features of MPS II include corneal opacification, glaucoma, retinopathy, optic nerve swelling or compression, and strabismus. All these complications occur as a result of the accumulation of GAGs in these ocular tissues [4,5].

Although several cases of Hunter syndrome with retinal findings suggestive of retinitis pigmentosa [6-8] have been reported, those of patients with maculopathy are rare.

## Case presentation

A 53-year-old male patient of East Asian descent with Hunter syndrome presented with a five-year history of inability to track moving objects and a three-year history of difficulty in recognizing colours. He denied any nyctalopia or photophobia. Genetic screening carried out 25 years prior showed that he was R48P hemizygous for the IDS gene. As part of his systemic condition, he had aortic valve replacement, severe mitral valve stenosis, and moderately severe obstructive lung function. He had been on Elaprase enzyme replacement therapy for the past 12 years, with his urine GAG being 3.4 mg/mmol at the time of ocular review.

His visual acuity was 6/30 for the right eye and 6/45 for the left eye, with intraocular pressures of 17 mmHg in each eye. He was unable to see anything on the test plates with Ishihara or Hardy-Rand-Rittler testing.

**Figure 1:**
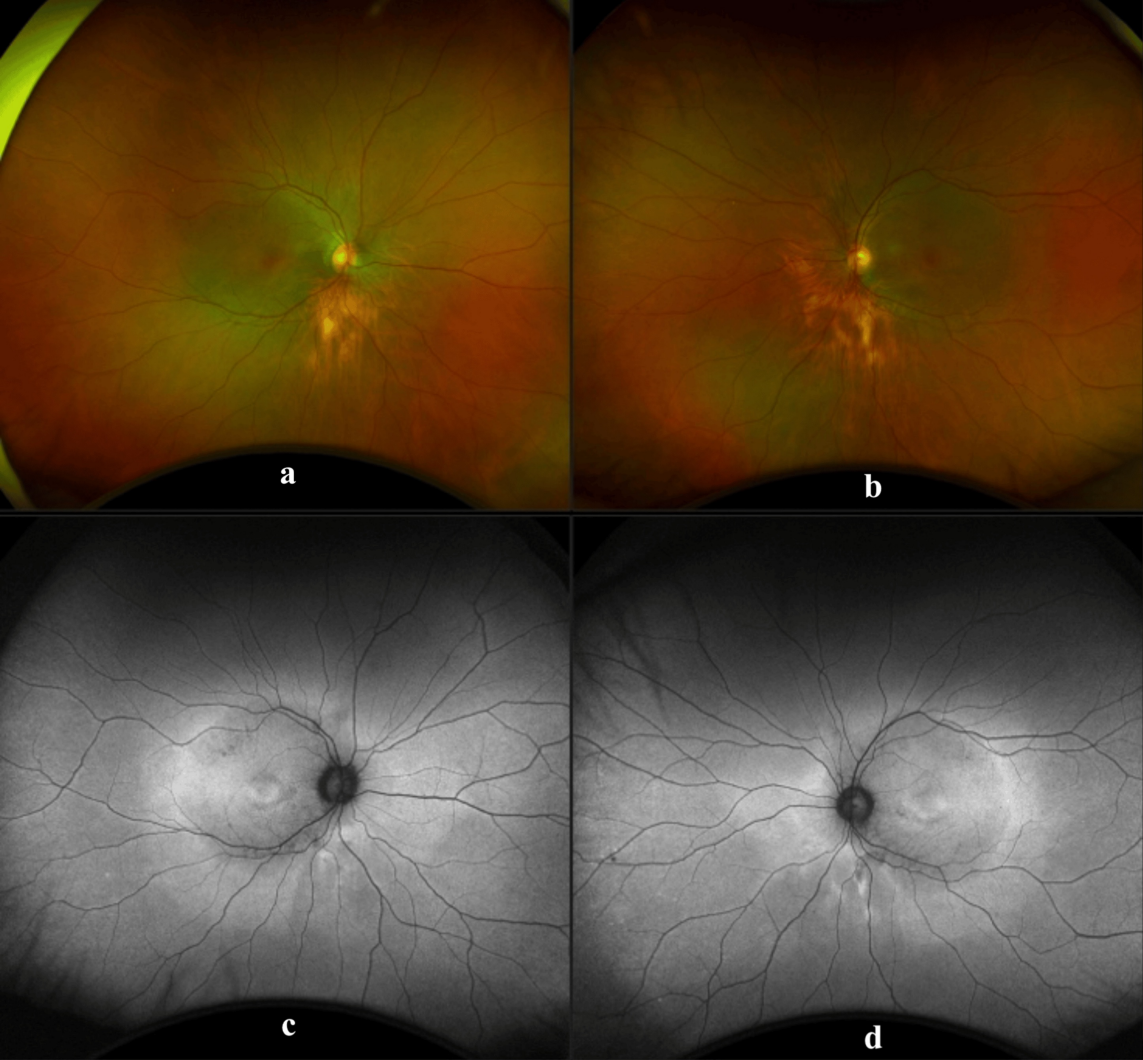
Widefield fundus imaging (a and b) and fundus autofluorescence (c and d) of the right and left eye Images taken using Optos (Optos plc, Dunfermline, UK) shows a posterior pole hyperautofluorescent ring in line with the vascular arcades and hypoautofluorescence at the macula area depicting a bull’s eye maculopathy.

His anterior segment examination was within normal limits; specifically, there was no corneal oedema in either eye. Posterior examination of both eyes revealed chorioretinal atrophy in the inferior quadrant of the right eye and the inferior nasal region of the left eye. There were no mid-peripheral bone spicules. The optic disc, the vessels, and the rest of the retina were within normal limits (Figures 1a, 1b). 

Ultrawide fundus photographs and ultrawide fundus autofluorescence photographs were obtained (Optos plc, Dunfermline, UK). Autofluorescence of both eyes showed a ring of hypoautofluorescence at the macula of both eyes (Figures 1c, 1d).

**Figure 2:**
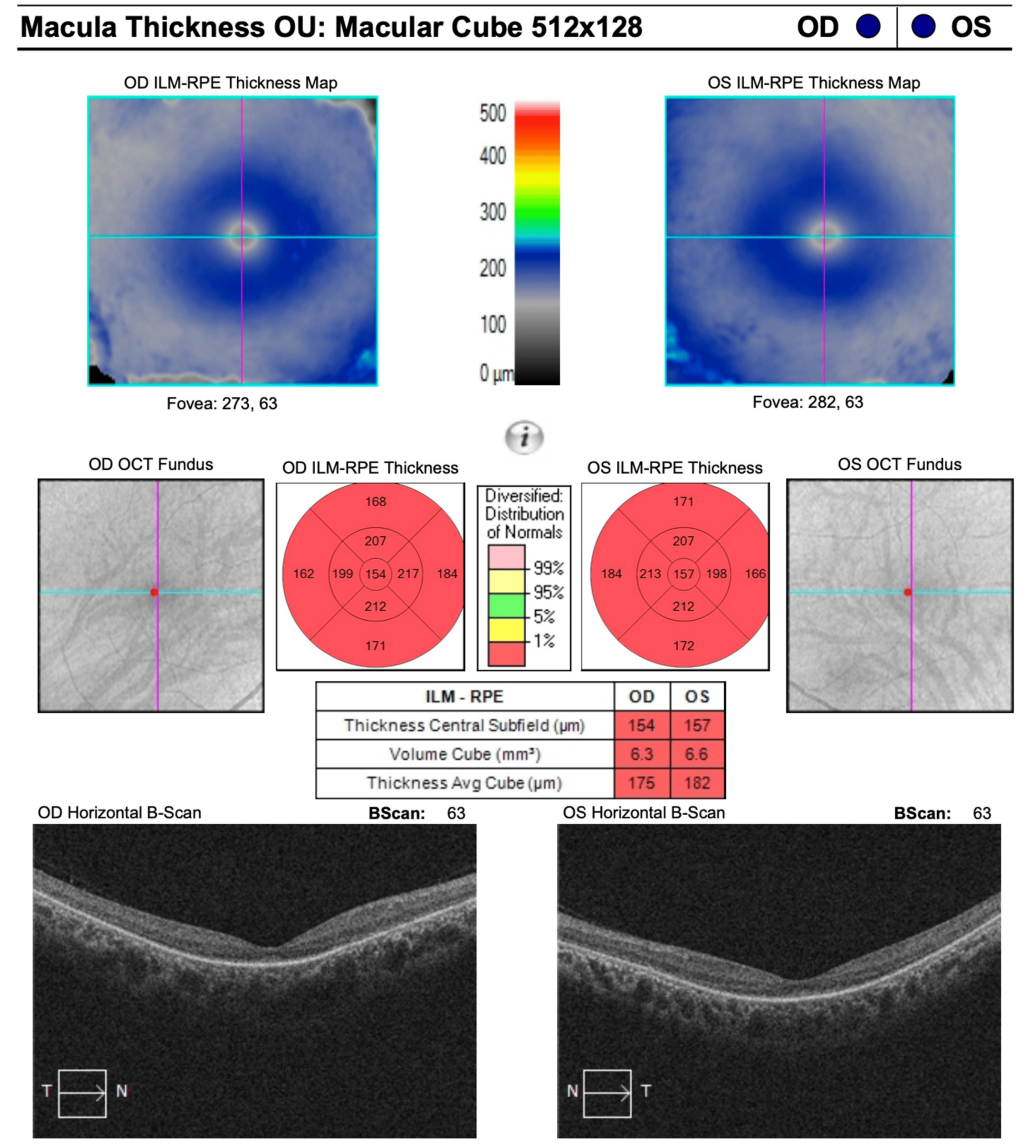
SD-OCT of the right and left eyes which showed severe thinning of the outer retina layers of the macula SD-OCT: Spectral-domain Optical Coherence Tomography

Spectral-domain Optical Coherence Tomography (SD-OCT) showed severe outer retinal layer attenuation, sparing a small island at the fovea in both eyes (Figure 2, Zeiss Cirrus, Carl Zeiss Meditec, Dublin, CA, USA).

**Figure 3:**
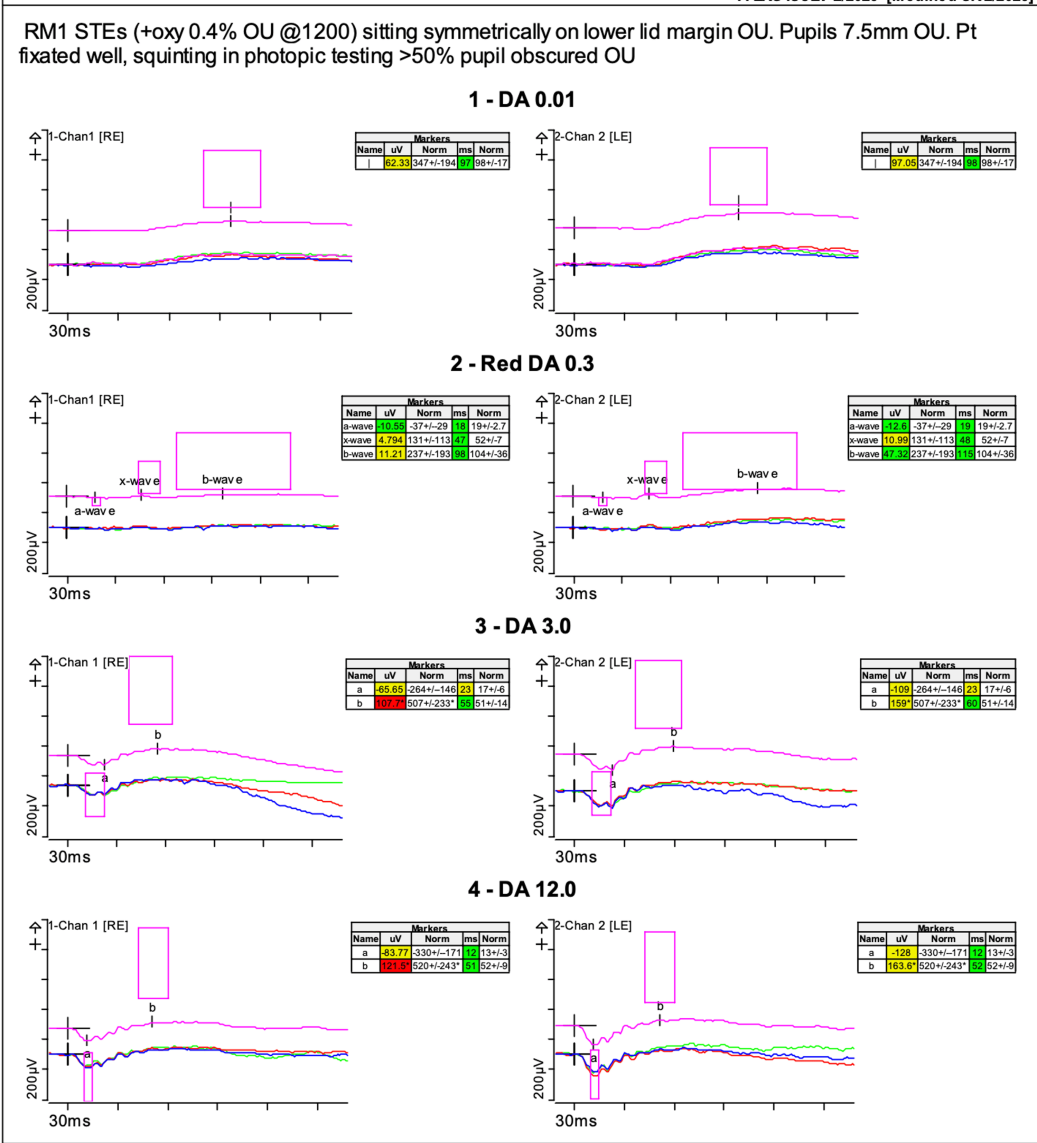
Full-field electroretinogram: scotopic responses shows mildly reduced rod-system function in both eyes The DA (Dark adapted) 0.01 shows well-defined b-waves in both eyes but a slight reduction in amplitude on the right. The red adaptation in DA 0.3 gave reduced reduction of x-wave results in both eyes. The dark adapted bright flash DA 3.0 also shows reduced a-wave and b-wave amplitudes. The pink box highlights the age matched ‘normal’ database responses.

**Figure 4:**
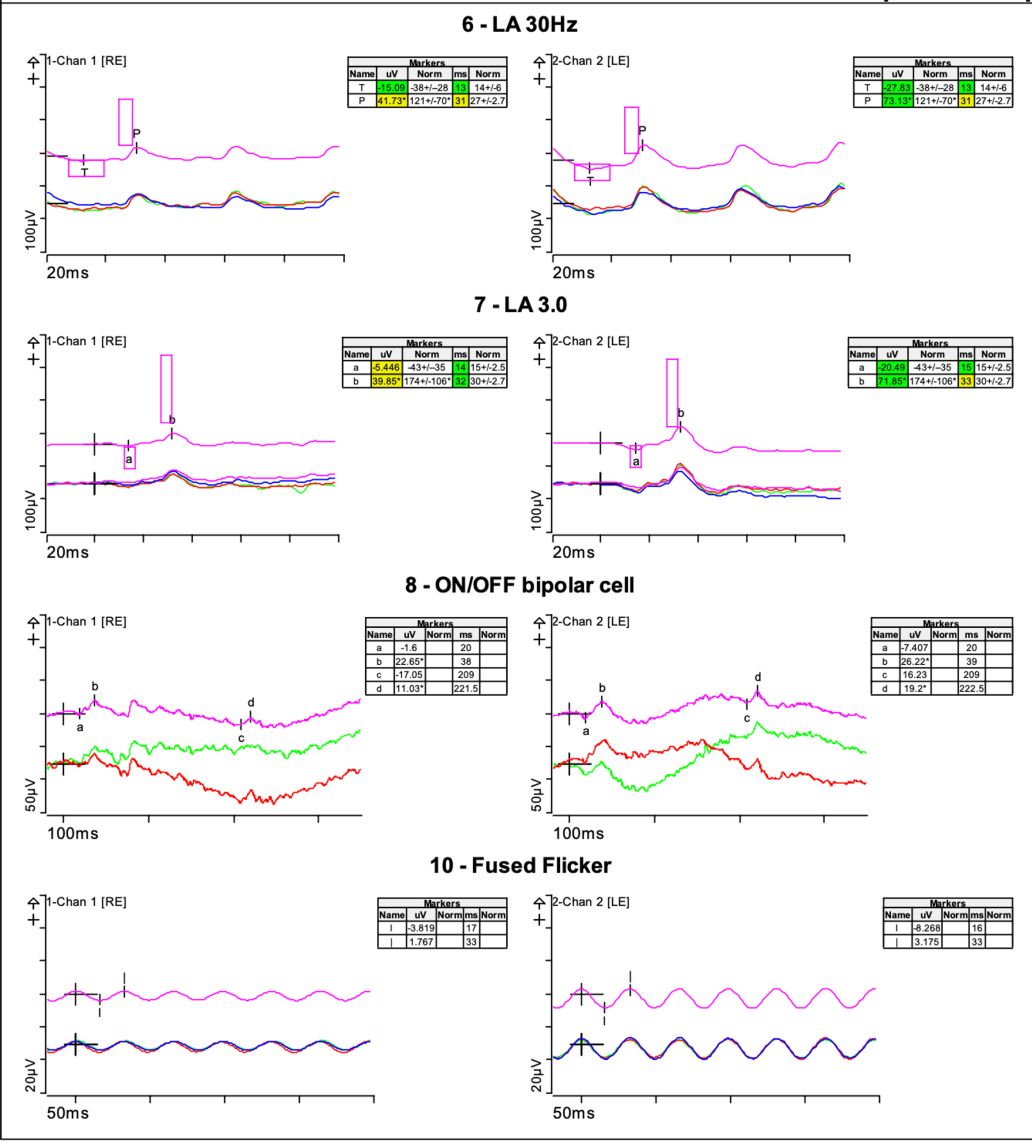
Full-field electroretinogram Photopic responses were also delayed and reduced as demonstrated in the Light adapted (LA) 30Hz and LA 3.0 responses. The pink box highlights the age matched ‘normal’ database responses.

Electrophysiological testing was performed using Espion (Diagnosys, Lowell, Massachusetts USA) according to the International Society for Clinical Electrophysiology of Vision (ISCEV) standards [9]. Full-field ERG (electroretinogram) was recorded using Dawson, Trick, and Litzkow (DTL) electrodes (Figures 3, 4).

The rod-specific ERG b-wave amplitudes were 125 microvolts in the right eye and 175 microvolts in the left eye. Red stimulation under dark adaptation gave reduced x-wave results. Dark-adapted bright flash a-wave and b-wave amplitudes were 110 and 165 microvolts in the right eye and 170 and 225 microvolts in the left eye. This represents mildly reduced rod-system function. Photopic responses were delayed and reduced with 30 Hertz (Hz) responses of 41.73 microvolts at 31 milliseconds (ms) in the right eye and 73.13 microvolts at 31 ms in the left eye.

**Figure 5:**
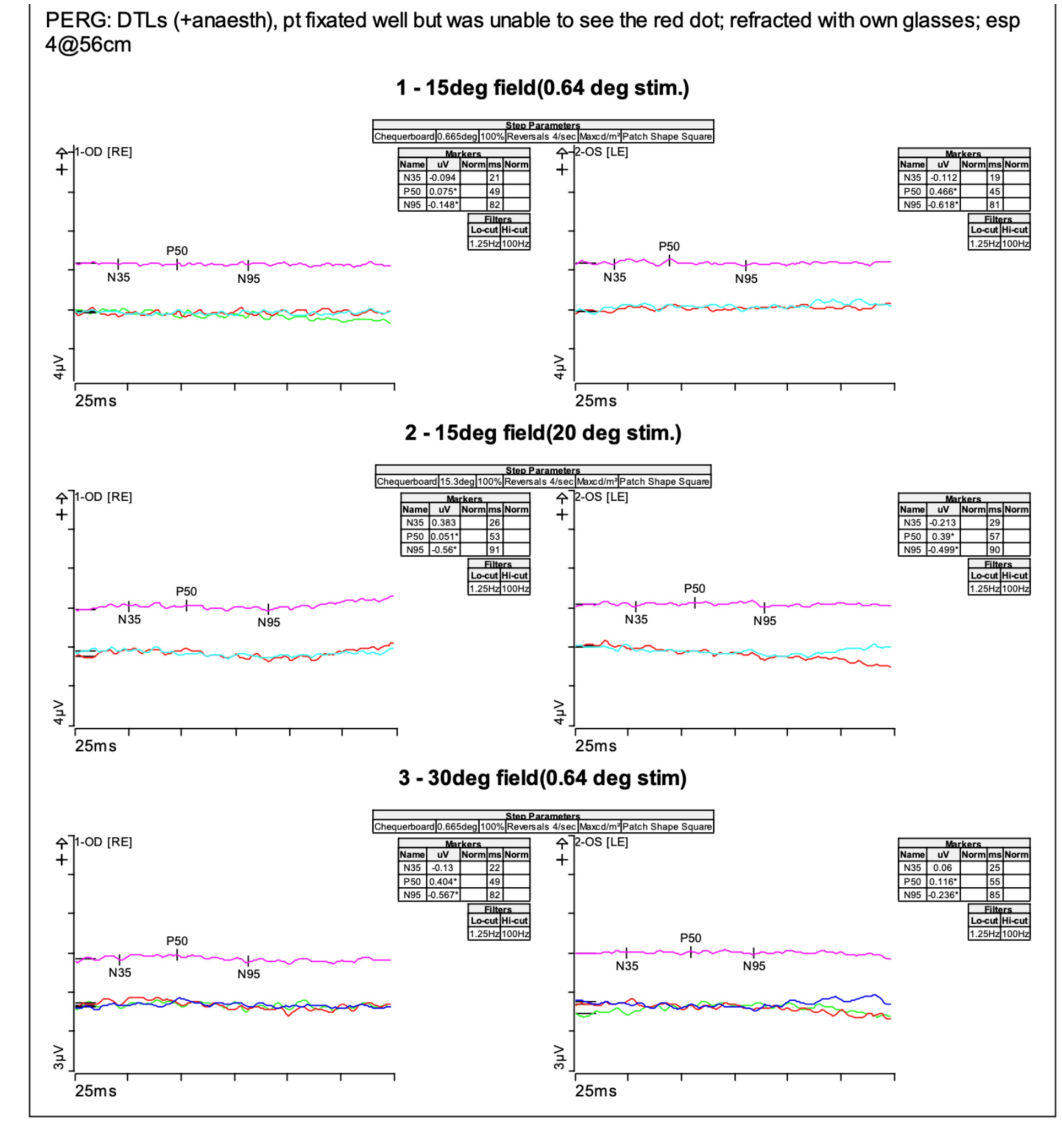
Pattern ERGs with the P50 responses severely attenuated in each eye to the 15-degree stimulus There was inadequate doubling of the responses to the 30-degree stimulus. ERG: electroretinogram

Pattern ERGs (Figure 5) were also recorded using DTL electrodes with the patient fixating well.

**Figure 6:**
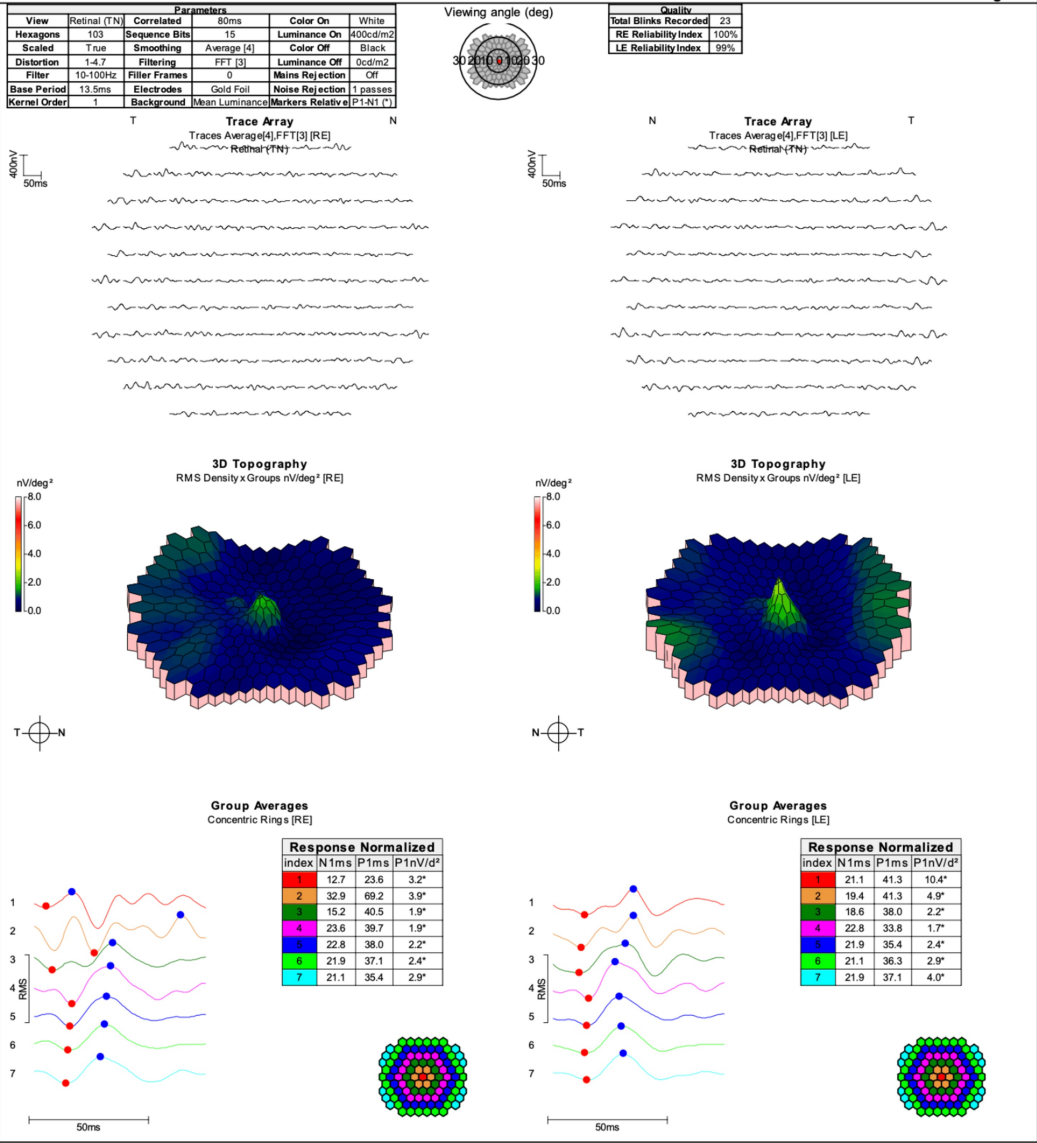
Multifocal ERG of the right and left eyes showed severely attenuated traces across the tested area ERG: electroretinogram

To the 15-degree stimulus, the P50 responses were severely attenuated in each eye and there was inadequate doubling of the responses to the 30-degree stimulus for the 30-degree field. This demonstrated reduced macular function. Multifocal ERG showed severely attenuated traces across the tested area in both eyes (Figure 6). 

**Figure 7:**
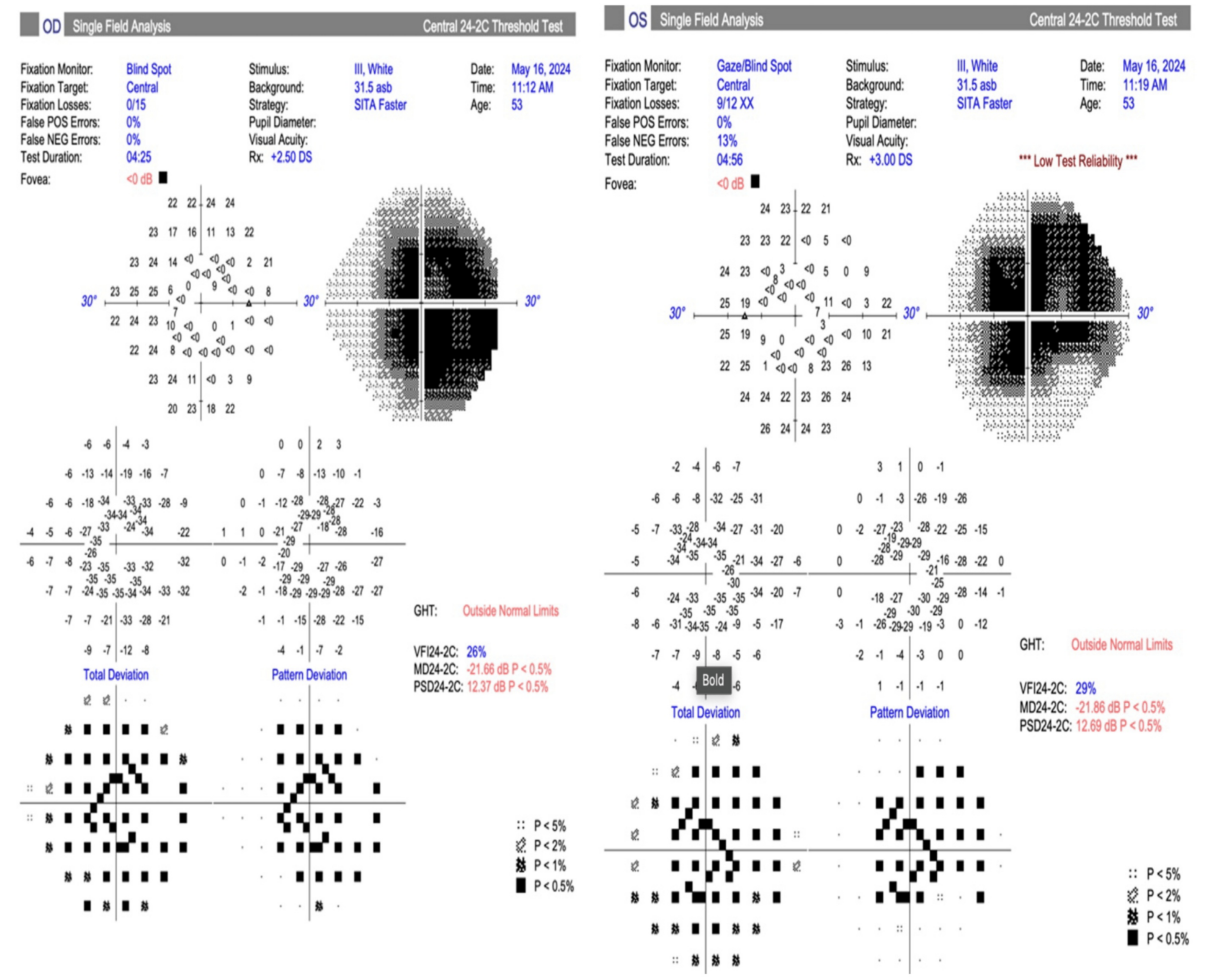
Humphrey visual fields of both eyes showed central scotoma which was worse on the right

As expected, the visual field test showed central scotoma in both eyes (Figure 7).

## Discussion

Hunter syndrome is the only X-linked recessive disease among the mucopolysaccharidoses. The genetic locus is Xq28, with the gene encoding for IDS, a lysosomal enzyme [10]. Patients with MPS are unable to degrade GAGs due to a lack of these lysosomal enzymes. GAGs are oligosaccharide components of proteoglycans. The accumulation of GAGs leads to thickening of tissues and compromises cell and organ functions. Clinical manifestations of the Hunter syndrome include coarse facial appearance, musculoskeletal defects, conductive and sensorineural hearing impairment, valvular heart disease, pulmonary hypertension, intellectual impairment and hydrocephalus [1].

MPS II phenotypically presents as two subtypes: MPS IIA and MPS IIB, with MPS IIB being a milder form of the disease. Patients with MPS IIB usually do not have any intellectual impairment [6,8]. In MPS IIA, patients present early with hydrocephalus, changes in behaviour and central nervous system involvement [2].

Several ophthalmic features distinguish Hunter syndrome from the other forms of MPS. Patients with Hunter syndrome usually have clear corneas [1]. This patient had no corneal oedema or corneal opacities, a feature which is consistent with findings in literature [1,11,12]. As most people with this condition have clear corneas, their visual acuity is usually better compared to other MPS subtypes.

Another ophthalmic feature of Hunter syndrome, which has serious consequences on the visual acuity, is retinal dystrophy. Retinal dystrophy in Hunter syndrome results from the deposition of GAGs in the retinal pigment epithelium and interphotoreceptor matrix, leading to the loss of photoreceptors [13]. Most patients reported in the literature have retinitis pigmentosa/rod-cone dystrophy. This is characterized by bone spicules, attenuated vessels, and waxy discs with preservation of central vision till the late stages of the condition [14,15]. On electrophysiological tests, rod-mediated responses are more severely attenuated than cone responses in patients with Hunter syndrome [8,16]. Our patient had none of these retinal signs on fundal examination. His presentation was that of bull’s eye maculopathy with loss of central vision, as correlated with his visual fields results. Maculopathy has been reported in a few cases of Hunter syndrome [17-20]. Bilateral cystoid macula oedema has been reported in a number of patients [19,21]. Macula oedema in these patients does not show petaloid leakage on fundus fluorescein angiography, suggesting neuroretinal degeneration rather than macula oedema [19]. Our patient did not have cystic spaces in the macula. 

The severe attenuation of the outer retinal layers corresponds to dysfunction of rods and cones as depicted on the pattern and multifocal electrophysiological tests. It is interesting to note that he did not exhibit the typical symptoms and fundal signs associated with rod dysfunction. This may be due to the preservation of rods in the peripheral fundus. It is also worth noting that our patient’s retinal degradation was not halted despite being on enzyme replacement therapy for the past 12 years. This is evident by the fact that he started experiencing his visual symptoms seven years after his first enzyme replacement therapy. This observation is likely attributable to the suboptimal permeability of the blood-retinal barrier to enzyme replacement therapy [22].

## Conclusions

We present a 54-year-old man with Hunter syndrome who has bull’s eyes maculopathy. Accumulation of GAGs in the retinal pigment epithelium resulted in photoreceptor loss affecting both rods and cones. His retinal dystrophy progressed despite being on enzyme replacement therapy. Current enzyme replacement therapies may not be effective in preventing retinal dystrophy in patients with Hunter syndrome due to their poor penetration of the blood-retinal barrier.

This case highlights the importance of regular ophthalmic monitoring and evaluation for individuals with Hunter syndrome. It also underscores the growing need for targeted ocular therapies in patients with Hunter syndrome to help preserve their vision.
